# Supplementary material for: Patterns of Intron Gain and Loss in Fungi
Source: PLoS Biol. 2004 Nov 30;2(12):e422. doi: 10.1371/journal.pbio.0020422 (PMC532390; doi:10.1371/journal.pbio.0020422)
Supplement: Table S1 — Also available at http://genes.mit.edu/NielsenEtAl/. (4.3 MB ZIP). [file pbio.0020422.st001.zip › NielsenEtAl/html/119.html]

AN8828.1.NCU06176.1.MG06435.1.FG06941.1


```
 CLUSTAL W (1.82) Multiple Sequence Alignments - Introns Inserted


Sequence 1: NCU06176.1	180 aa
Sequence 2: FG06941.1	145 aa
Sequence 3: MG06435.1	148 aa
Sequence 4: AN8828.1	167 aa
Alignment Length: 185 aa
Number Identitical Residues: 85 aa
Alignment Score (without introns) 4124


MG06435.1 	MVVYSFYIFDRHT1ECIYTKQWLPPPPS--------------------------------
NCU06176.1	MVIYSFYIFDRHT1ECIYSKTWLPPPPAPGAQPQHDISTSTTGAAVASSDPHHHNPNTLR
FG06941.1 	MVVYSFHIFDRHT1ECVYSKSWLPS-----------------------------------
AN8828.1  	MTVYSFYIFDRHA1ECIYKRRWLPRPASIVGKSSRPTS------------------DTLT
          	*.:***:*****: **:*.: *** ..:  ....   :                  .:  

MG06435.1 	APDTGKPGRDSRK----DDAKLIFGTVFSLRNMVRKLGGDDDA~FIAYRTAQYKLHFYET
NCU06176.1	SSRSAVSAANKAS----DDAKLIFGTVFSLRNMVRKLGGEDDA~FISYRTAQYKLHYYET
FG06941.1 	SAGSEVAAPTTSS----DDAKLLFGTVFSLRNMVRKLGGDDDA~FISYRTAQYKLHFYET
AN8828.1  	AANGIAPVPNQSARSTDDDAKLIFGTVFALRNMVRKLGGEDDN2FVTYRTSQYKLHYYET
          	:.    .       ::.*****:*****:**********:**  *::***:*****:***

MG06435.1 	PTNLRFVLLTDTASMSMRNVLHQIYINLWVEYV1VKNPLAPVEHKGGEGVKNELFELGLD
NCU06176.1	ASNLRFVMLTDTGTLSMRNVMHQIYINLWCEYV1VKNPLAPVEHKGGAGVRNELFELGLN
FG06941.1 	PANLRFVILTDTATLSMRNVLHQIYINLWVEYV1VKNPLAPVEHKKGEGVKNELFELGLD
AN8828.1  	LTNIKFVMITDVKSPSMRVALQQIYINLYVEYV1VKNPLSPTEHPGGVGVNNELFEQSLE
          	 :*::**::**. : *** .::******: *** *****:*.**  * **.***** .*:

MG06435.1 	QFI~RGLM-
NCU06176.1	QFI~TGLM-
FG06941.1 	QFI~RGLM-
AN8828.1  	QFV0TRVLA
          	**:   :::
```
